# MicroRNA‐148a‐3p suppresses epithelial‐to‐mesenchymal transition and stemness properties via Wnt1‐mediated Wnt/β‐catenin pathway in pancreatic cancer

**DOI:** 10.1111/jcmm.15900

**Published:** 2020-10-07

**Authors:** Xiaowei Fu, Le Hong, Zhengjiang Yang, Yi Tu, Wanpeng Xin, Ming Zha, Shuju Tu, Gen Sun, Yong Li, Weidong Xiao

**Affiliations:** ^1^ Department of General Surgery The First Affiliated Hospital of Nanchang University Nanchang Jiangxi China; ^2^ Department of Pathology The First Affiliated Hospital of Nanchang University Nanchang Jiangxi China

**Keywords:** epithelial‐to‐mesenchymal transition, miR‐148a‐3p, pancreatic cancer, stemness properties, Wnt1

## Abstract

Although miR‐148a‐3p has been reported to function as a tumour suppressor in various cancers, the molecular mechanism of miR‐148a‐3p in regulating epithelial‐to‐mesenchymal transition (EMT) and stemness properties of pancreatic cancer (PC) cells remains to be elucidated. In the present study, we demonstrated that miR‐148a‐3p expression was remarkably down‐regulated in PC tissues and cell lines. Moreover, low expression of miR‐148a‐3p was associated with poorer overall survival (OS) in patients with PC. In vitro, gain‐of‐function and loss‐of‐function experiments showed that miR‐148a‐3p suppressed EMT and stemness properties as well as the proliferation, migration and invasion of PC cells. A dual‐luciferase reporter assay demonstrated that Wnt1 was a direct target of miR‐148a‐3p, and its expression was inversely associated with miR‐148a‐3p in PC tissues. Furthermore, miR‐148a‐3p suppressed the Wnt/β‐catenin pathway via down‐regulation of Wnt1. The effects of ectopic miR‐148a‐3p were rescued by Wnt1 overexpression. These biological functions of miR‐148a‐3p in PC were also confirmed in a nude mouse xenograft model. Taken together, these findings suggest that miR‐148a‐3p suppresses PC cell proliferation, invasion, EMT and stemness properties via inhibiting Wnt1‐mediated Wnt/β‐catenin pathway and could be a potential prognostic biomarker as well as a therapeutic target in PC.

## 1 INTRODUCTION

Pancreatic cancer (PC) is one of the most aggressive cancers representing the fourth leading cause of cancer‐related death worldwide, with an approximately 5‐year survival rate of 9%.^1^ Despite chemotherapy is currently an indispensable therapeutic approach for PC both in the adjuvant setting and in the metastatic setting, its efficacy still remains unsatisfactory.^2^ MicroRNAs (miRNAs) are small non‐coding RNAs consisting of approximately 18‐22 nucleotides, which play crucial roles in the post‐transcriptional regulation of target genes linked to their 3′‐untranslated regions (3'UTR). Increasing scientific evidence indicates that miRNAs participate in various physiological and pathological processes, including cell proliferation, differentiation, metabolism and tumorigenesis.^3^, ^4^ As a member of the miR‐148/152 family, miR‐148a has been reported to function as a tumour suppressor in various human cancers, including hepatocellular carcinoma,^5^ cholangiocarcinoma,^6^ gastrointestinal cancers^7^, ^8^ and PC.^9^, ^10^ However, the underlying molecular mechanism of miR‐148a‐3p in PC remains to be elucidated.

Recently, accumulating evidence implicates that both epithelial‐to‐mesenchymal transition (EMT) phenotype cells and cancer stem cells (CSCs) are associated with the acquisition of tumour malignant behaviour.^11^, ^12^, ^13^ EMT is a process from epithelial cell phenotype to mesenchymal cell phenotype, resulting in enhanced migration and invasion capacity, which plays an important role in tumorigenesis and evolution^11^, ^14^; CSCs represent a small subpopulation of cells within tumours, which exhibit the ability to self‐renew, and contribute to cell growth, tumorigenicity, drug resistance, tumour relapse and metastasis.^15^, ^16^, ^17^, ^18^ Notably, cancer cells may gain CSC‐like properties through EMT, and CSCs exhibit an EMT phenotype for metastasis.^19^ Overlapping of these two properties implies that they may share similar molecules/pathways. To date, multiple miRNAs have been proved to be involved in EMT and the maintenance of CSC‐like properties. Yan et al^20^ reported that miR‐148a inhibits the metastasis of hepatocellular carcinoma by blocking the EMT process and CSC‐like properties. Also, miR‐148a expression is significantly attenuated in cancer stem cell–like hepatocellular carcinoma subtype.^21^ However, the role of miR‐148a‐3p in EMT and the maintenance of stemness properties in PC cells have not yet been clarified.

As a member of the Wnt ligand gene family, Wnt1 aberrantly activates the Wnt/β‐catenin pathway in many human tumours, regulates the transcription of downstream genes and then affects cell proliferation, EMT, metastasis and the maintenance of stemness properties.^22^, ^23^, ^24^ In our study, We firstly demonstrated that Wnt1 was a direct functional target of miR‐148a‐3p, and miR‐148a‐3p suppressed cell proliferation, invasion, EMT and stemness properties via Wnt1‐mediated Wnt/β‐catenin pathway in PC.

## 2 MATERIALS AND METHODS

### 2.1 PC tissue specimens

From January 2013 to December 2017, 61 paired PC and corresponding adjacent non‐tumorous tissues (ANT) were obtained from patients pathologically diagnosed with pancreatic ductal adenocarcinoma (PDAC), who underwent surgical resection at the First Affiliated Hospital of Nanchang University, China. Patients enrolled in our study received neither chemotherapy nor radiotherapy prior to surgery. Written informed consent was obtained from each participant in accordance with the Declaration of Helsinki. The present study was approved by the Ethics Committee of the First Affiliated Hospital of Nanchang University.

### 2.2 Cell lines and cell culture

The normal human pancreatic ductal epithelial cell line HPDE and human PC cell lines Panc‐1, SW1990, Mia PaCa‐2 and BxPC‐3 were obtained from the Type Culture Collection of the Chinese Academy of Sciences. The human PC cell line Capan‐2 was obtained from the ATCC. These cell lines were routinely maintained in DMEM (Invitrogen) supplemented with 10% FBS in a humidified atmosphere containing 5% carbon dioxide at 37°C.

### 2.3 RNA extraction and quantitative real‐time PCR

RNA was isolated from snap‐frozen fresh specimens and cell lines using TRIzol Reagent (Thermo Fisher Scientific) and reverse‐transcribed using the PrimeScript RT Reagent Kit (Takara), according to the manufacturer’s instructions. Then, quantitative PCR was performed using SYBR Premix Ex Taq (Takara), following the manufacturer’s instructions. U6 and GAPDH were regarded as the internal control, and the relative gene expression was determined using the $2^{‐\Delta \Delta C_{t}}$ method. The primer sequences are listed in Table 1.

**TABLE 1:** qPCR primers used in this study

Genes	Primers	Sequences (5′‐3′)
*miR‐148a‐3p*	Forward	TGCGGTCAGTGCACTACAGAAC
	Reverse	CCAGTGCAGGGTCCGAGGT
*Wnt1*	Forward	GAGCCACGAGTTTGGATGTT
	Reverse	TGAGGAGAGAAGAGGGACCA
*β‐catenin*	Forward	CATCTACACAGTTTGATGCTGCT
	Reverse	GCAGTTTTGTCAGTTCAGGGA
*MMP‐9*	Forward	GCGAGGTGCGCCTTAGTC
	Reverse	CGGTGCCGGATAGTCCACAGGA
*Cyclin D1*	Forward	GCATCTACACCGACAACTCCATC
	Reverse	CGTGTGAGGCGGTAGTAGGA
*E‐cadherin*	Forward	GACAACAAGCCCGAATT
	Reverse	GGAAACTCTCTCGGTCCA
*N‐cadherin*	Forward	GTATCCGGTCCGATCTGCA
	Reverse	ATAGTCCTGCTCACCACCAC
*Vimentin*	Forward	ATTCCACTTTGCGTTCAAGG
	Reverse	CTTCAGAGAGAGGAAGCCGA
*CD133*	Forward	CAGAAGGCATATGAATCC
	Reverse	CACCACATTTGTTACAGC
*Nanog*	Forward	AAGAACTCTCCAACATCCTGAAC
	Reverse	CCTTCTGCGTCACACCATT
*Oct4*	Forward	ATTCAGCCAAACGACCATCT
	Reverse	TCTCACTCGGTTCTCGATACTG
*Sox2*	Forward	GCCGAGTGGAAACTTTTGTCG
	Reverse	GGCAGCGTGTACTTATCCTTCT
*U6*	Forward	CTCGCTTCGGCAGCACA
	Reverse	AACGCTTCACGAATTTGCGT
*GAPDH*	Forward	CATCACCATCTTCCAGGAGCG
	Reverse	TGACCTTGCCCACAGCCTTG

### 2.4 Western blotting

Cells were lysed using RIPA buffer (Beyotime) containing protease inhibitors. Protein concentrations were detected using a Pierce BCA Protein Assay Kit (Thermo Fisher Scientific). Protein samples were separated by 10% SDS‐PAGE, transferred to PVDF membranes and then incubated with primary antibodies at 4°C overnight and secondary antibodies for 2 hours at room temperature. Finally, the proteins were visualized with enhanced chemiluminescence reagents (Millipore). The primary antibodies against β‐catenin (#8480, 1:1000), MMP‐9 (#13667, 1:1000), cyclin D1 (#2978, 1:500), E‐cadherin (#14472, 1:1000), N‐cadherin (#13116, 1:1000), vimentin (#5741, 1:1000), CD133 (#86781, 1:1000), Nanog (#4903, 1:2000), Oct4 (#2750, 1:1000) and Sox2 (#3728, 1:1000) were obtained from Cell Signaling Technology, and the antibodies against Wnt1 (ab15251, 1:1000) and GAPDH (ab8245, 1:2000) were obtained from Abcam. The relative protein levels were analysed by using ImageJ software.

### 2.5 Lentivectors and plasmid transfection

Lentivirus encoding miR‐148a‐3p, anti‐miR‐148a‐3p and their respective negative control were synthesized by GenePharma and then infected Mia PaCa‐2, BxPC‐3 and Capan‐2 cells. The infection efficiency was confirmed by quantitative real‐time PCR (qRT‐PCR). For generation of stably transfected cells, the cells were treated with 2 μg/mL of puromycin (Sigma) for 2 weeks, and then, GFP‐positive cells were selected for subsequent assays. Special siRNA against Wnt1 (si‐Wnt1), Wnt1 overexpression plasmid (Wnt1‐pcDNA3.1‐EGFP) and their respective negative control vector were purchased from GenePharma and then transfected using Lipofectamine 3000 (Invitrogen). The transfection efficiency was confirmed by qRT‐PCR. In the specified group, cells were exposed to 10 ng/mL TGF‐β (R&D Systems) and then incubated at 37°C for 48 hours.

### 2.6 CCK‐8 assay and colony formation assay

For CCK‐8 assay, cells with different treatments were seeded in 96‐well plate with a density of 5 × 10^3^ cells per well. At the indicated time‐points (1, 2, 3, 4 and 5 days), 10 μL CCK‐8 reagent was added to each well and incubated in dark at 37°C for another 1 hour. Finally, the spectrophotometric absorbance at 450 nm was measured for each well. For colony formation assay, cells with different treatments were seeded in 6‐well plate with a density of 1 × 10^3^ cells per well. After incubation for 14 days, the cells were fixed with 4% paraformaldehyde and then stained with 0.5% crystal violet. Finally, individual clones were counted to evaluate cell proliferation. Individual clones (>50 cells/clone) were counted. At least three wells were assessed for each group, and the mean was calculated.

### 2.7 Wound healing assay

Cells with different treatments were seeded in a 6‐well plate and cultured to grow to confluence. Then, the monolayer was scraped with a pipette tip (200 μL) to produce an artificial wound gap. Finally, the wound closure was photographed after 48‐h incubation using an inverted microscope (Olympus), and the wound closure rate was calculated by using ImageJ software.

### 2.8 Cell migration and invasion assay

The migration and invasion capacities of cells were detected by Transwell assay according to manufacturer’s instruction as described previously.^10^ Briefly, in the invasion assay, cells were added to the Transwell chambers pre‐coated with Matrigel (BD Biosciences). Then, the chambers were placed in a 24‐well plate for incubation 24 hours. Fixed with 4% paraformaldehyde and stained with 0.1% crystal violet, the number of invaded cells was counted under a microscope (Olympus) in five fields with random choice. The same steps were performed in the cell migration assay; however, Matrigel was not used. The experiment was repeated three times.

### 2.9 Flow cytometry analysis and magnetic cell sorting

Cells with different treatments were resuspended at 1 × 10^6^ cells per mL in PBS containing 2% FBS and then subsequently stained with PE‐conjugated CD133 antibody (BioLegend) for 30 minutes on ice in dark. After washing with ice‐cold PBS, each sample was analysed by flow cytometry (BD Bioscience). The positive CD133 subpopulations of Capan‐2 cells were sorted from using MACS separation (Miltenyi Biotec) according to the manufacturer’s protocol as described previously.^25^

### 2.10 Sphere formation assay

Capan‐2 cells in different groups were seeded into 6‐well ultra‐low attachment cluster plates (Corning) at a density of 5 × 10^2^ cells per well, and then cultured in serum‐free DMEM/F12 (Invitrogen) supplemented with 1% N2 (Gibco), 2% B27 (Gibco), 20 ng/mL of basic fibroblast growth factor (Gibco), 20 ng/mL of epidermal growth factor (Gibco) and 5 μg/mL insulin (Sigma). After two weeks, the size and number of spheres were counted.

### 2.11 Dual‐luciferase reporter assay

The 3′‐UTR of Wnt1 mRNA containing the miR‐148a‐3p binding sequence (Wnt1 3′ UTR‐wt) or a mutant variant (Wnt1 3′ UTR‐mut) was amplified by PCR and cloned into the XbaI and NotI restriction sites of the pmirGLO Dual‐Luciferase miRNA Target Expression Vector (Promega). HEK‐293T cells were plated into 24‐well plates and cotransfected with either 100 nmol/L miR‐148a‐3p mimics or inhibitors (GenePharma), together with 100 ng pmirGLO Vector. After 48 hours, luciferase activities in transfected cells were detected using the Dual‐Luciferase Assay System (Promega) according to the manufacturer’s protocol. The firefly luciferase activity was measured and normalized based on Renilla luciferase activity. The experiments were performed independently in triplicate.

### 2.12 Xenograft experiments

Male BALB/c nude mice aged 5‐6 weeks were purchased from Hunan SJA Laboratory Animal Co. Ltd. Cells (1 × 10^7^ cells per mice) that stably expressed miR‐148a‐3p, anti‐miR‐148a‐3p or corresponding controls were subcutaneously injected into the mice, with six mice in each group. Tumour size was recorded weekly using the formula: width^2^ × length/2. Mice were killed 5 weeks after inoculation, and tumours were weighed. All animal experiments were performed in accordance with the experimental animal use guidelines of the National Institutes of Health and approved by the Ethics Committee for Animal Experiments of the First Affiliated Hospital of Nanchang University.

### 2.13 Immunohistochemistry assays

The formalin‐fixed paraffin‐embedded tissues of xenograft tumours were used for immunohistochemistry (IHC) analysis and performed as described previously.^26^ The antibodies against β‐catenin (#8480, 1:100), cyclin D1 (#2978, 1:50), Ki‐67 (#9027, 1:600), E‐cadherin (#14472, 1:100), vimentin (#5741, 1:200), CD133 (#86781, 1:500), Nanog (#4903, 1:500) and Oct4 (#2750, 1:200) were obtained from Cell Signaling Technology. The antibody against Wnt1 (ab15251, 1:100) was obtained from Abcam. The staining intensity and proportion of indicated proteins were evaluated and scored by two observers independently in a blinded manner using a microscope (Olympus).

### 2.14 Statistical analysis

Measurement data were shown as mean ± SD and analysed using SPSS 24.0 statistical software. Values were analysed using two‐tailed Student’s *t* test or one‐way ANOVA for most of the experiments. Mann‐Whitney *U* test was used to compare the expression of miR‐148a‐3p between PC tissues and their corresponding adjacent tissues. Spearman’s correlation test was used for correlation analyses. Survival analysis was performed using the Kaplan‐Meier method, and differences were assessed with the log‐rank test. The experimental data were representative of at least three independent experiments and were considered statistically significant at *P* < .05.

## 3 RESULTS

### 3.1 Low expression of miR‐148a‐3p is correlated with poor prognosis in patients with PC

**FIGURE 1:**
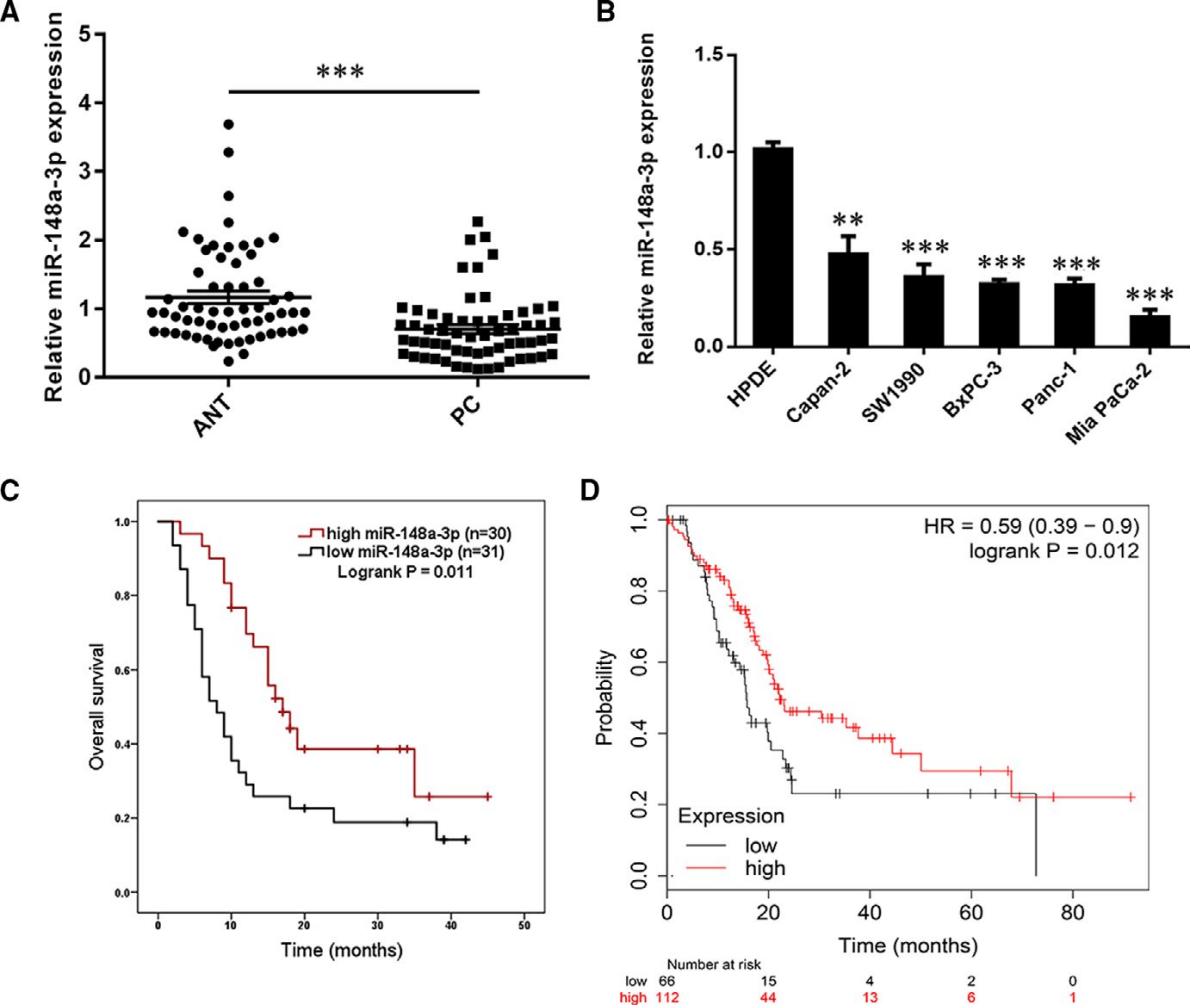
The expression of miR‐148a‐3p is down‐regulated in pancreatic cancer (PC) tissues and cell lines. A, miR‐148a‐3p expression was assessed in 61 pairs of PC and corresponding ANT tissues using RT‐qPCR. B, RT‐qPCR was used for the determination of miR‐148a‐3p expression in five PC cell lines (Panc‐1, SW1990, Mia Paca‐2, BxPC‐3 and Capan‐2) and the normal HPDE cell line. C, Kaplan‐Meier analysis of the correlation between miR‐148a‐3p expression and overall survival of PC patients (log‐rank test, *P* = .011). D, Data derived from the KM Plotter online database showed that low expression of miR‐148a‐3p was associated with poorer OS in patients with PC. Data were expressed as means ± SD of three independent experiments. **P < .01, ***P < .001

Our previous study has shown that miR‐148a was down‐regulated in 33 PC tissues, and miR‐148a expression was significantly associated with histological grade, tumour size, lymph node status and TNM stage.^9^ In the present study, we expanded these samples to 61 pairs of PC tissues. The qRT‐PCR results confirmed that miR‐148a‐3p was significantly decreased in PC tissues as compared with their corresponding adjacent tissues (Figure 1A). Consistently, miR‐148a‐3p was markedly down‐regulated in five PC cell lines (Panc‐1, SW1990, Mia Paca‐2, BxPC‐3 and Capan‐2) relative to normal HPDE cell line (Figure 1B). By dividing all patients into high‐ and low‐expression group based on the median relative expression level of miR‐148a‐3p, we further found that patients with lower expression of miR‐148a‐3p exhibited significantly poorer overall survival (OS; Figure 1C). Consistently, data derived from the KM Plotter online database (http://kmplot.com/analysis/) were in agreement with this result (Figure 1D). Together, these data revealed that miR‐148a‐3p was down‐regulated in PC, which was correlated with poor prognosis in patients with PC.

### 3.2 miR‐148a‐3p inhibits malignant behaviour of PC cells

**FIGURE 2:**
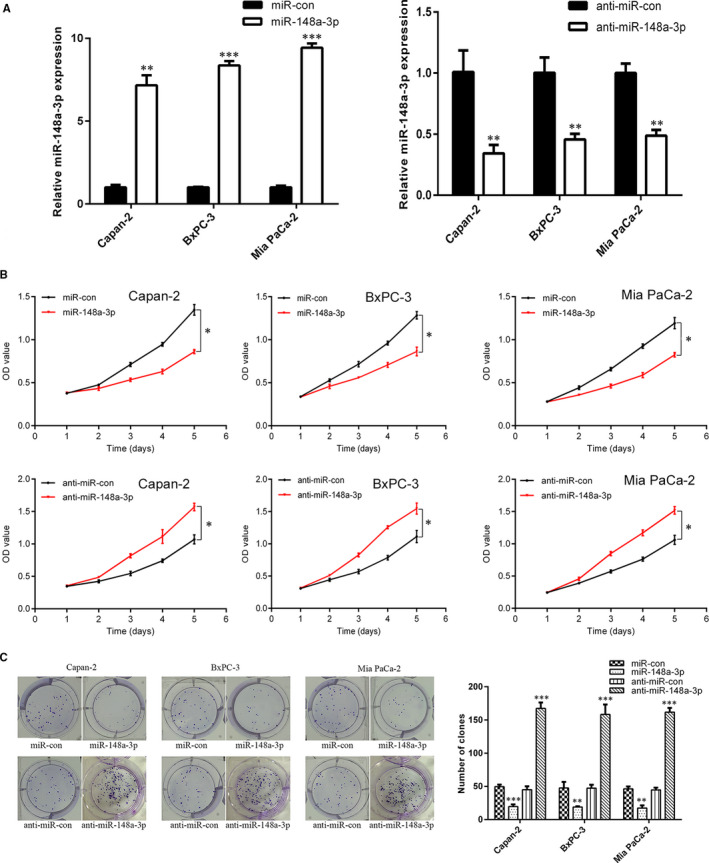
miR‐148a‐3p inhibits the proliferation of PC cells. A, After infecting lentivirus encoding miR‐148a‐3p or anti‐miR‐148a‐3p, the expression level of miR‐148a‐3p in Capan‐2, BxPC‐3 and Mia PaCa‐2 cells was confirmed using RT‐qPCR analysis. B,C, The regulatory roles of miR‐148a‐3p in PC cell proliferation and colony formation were evaluated using CCK‐8 assay and colony formation assay. Data were expressed as means ± SD of three independent experiments. *P < .05, **P < .01, ***P < .001

**FIGURE 3:**
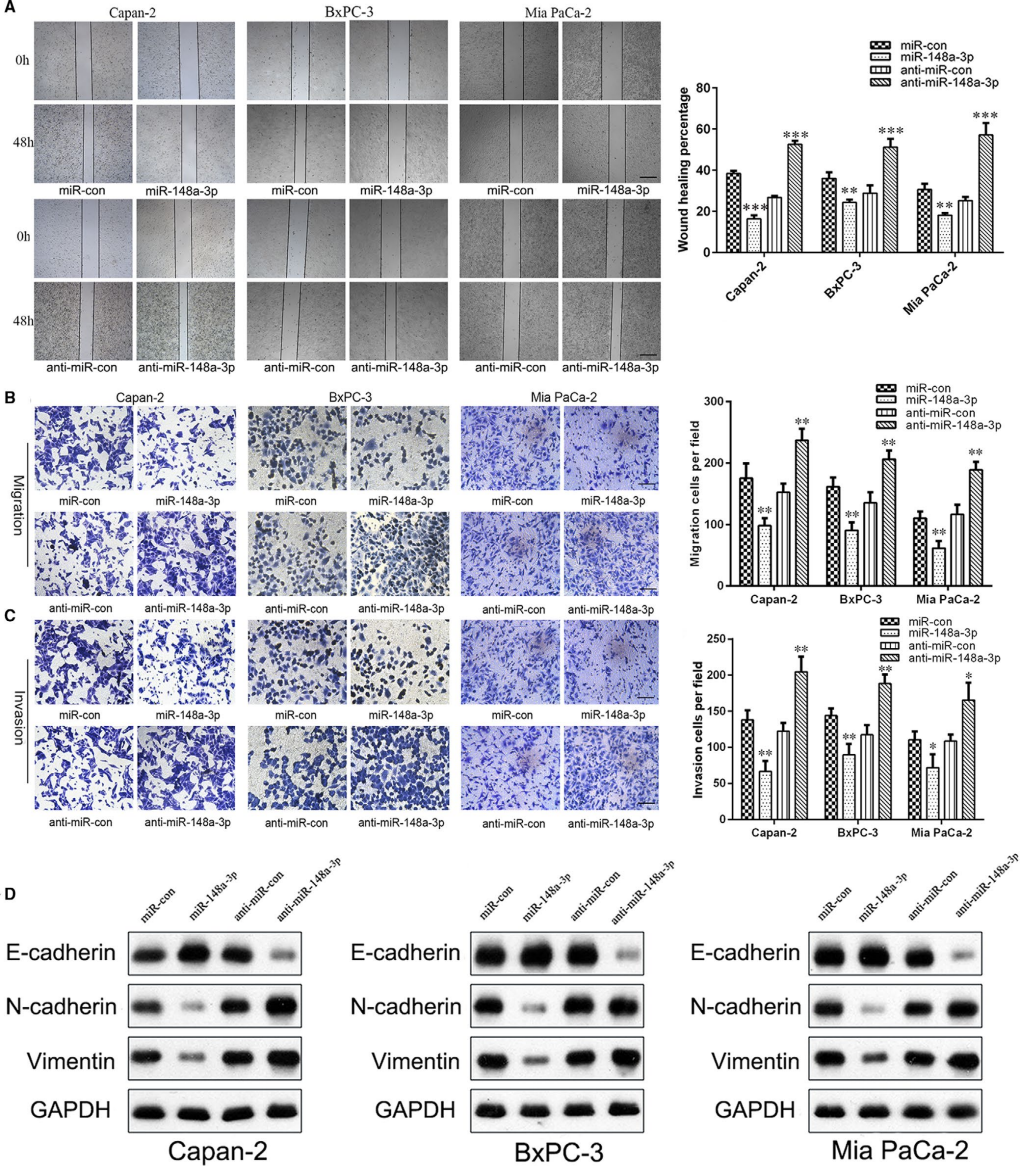
miR‐148a‐3p inhibits the migration and invasion of PC cells through inhibiting the EMT process. A, The regulatory roles of miR‐148a‐3p in Capan‐2, BxPC‐3 and Mia PaCa‐2 cell motility were evaluated using wound healing assay (40×). Scale bars, 250 μm. B,C, The regulatory roles of miR‐148a‐3p in Capan‐2, BxPC‐3 and Mia PaCa‐2 cell migration and invasion capacities were evaluated using Transwell assay (200×). Scale bars, 50 μm. D, The protein levels of EMT markers (E‐cadherin, N‐cadherin and vimentin) in Capan‐2, BxPC‐3 and Mia PaCa‐2 cells were detected using Western blot analysis. Data were expressed as means ± SD of three independent experiments. *P < .05, **P < .01, ***P < .001

As the Capan‐2, BxPC‐3 and Mia PaCa‐2 cell lines were obtained from the primary tumour, with different differentiation degrees^27^ and different expression levels of miR‐148a‐3p (Figure 1B), these three cell lines were selected for subsequent cell function investigation. Firstly, Capan‐2, BxPC‐3 and Mia PaCa‐2 cells that stably expressed miR‐148a‐3p, anti‐miR‐148a‐3p and the corresponding negative controls were established, respectively. The efficacy of infection was confirmed by qRT‐PCR (Figure 2A). Both the CCK‐8 assay and colony formation assay showed that miR‐148a‐3p overexpression significantly inhibited cell proliferation, while miR‐148a‐3p depletion increased cell proliferation of Capan‐2, BxPC‐3 and Mia PaCa‐2 cells (Figure 2B,C). To identify the function of miR‐148a‐3p on metastasis of PC, the migration and invasion capacities were detected by using the wound healing assay and Transwell assay. Wound healing assay indicated that miR‐148a‐3p depletion markedly enhanced the migration capacity, whereas miR‐148a‐3p overexpression repressed the migration capacity of Capan‐2, BxPC‐3 and Mia PaCa‐2 cells (Figure 3A). Transwell assay results demonstrated that miR‐148a‐3p depletion promoted the migration and invasion capacities, while miR‐148a‐3p overexpression significantly impaired the migration and invasion capacities of PC cells (Figure 3B,C). Taken together, these findings indicated that miR‐148a‐3p could inhibit the malignant behaviour of PC cells in vitro.

### 3.3 miR‐148a‐3p inhibits the EMT process of PC cells

It is well‐known that EMT plays an important role in promoting cell migration and invasion.^28^ In the present study, we further evaluated the expression of EMT markers (E‐cadherin, N‐cadherin and vimentin) by Western blotting in PC cells. Our results showed that the levels of mesenchymal markers N‐cadherin and vimentin were significantly up‐regulated, whereas epithelial marker E‐cadherin was down‐regulated in Capan‐2, BxPC‐3 and Mia PaCa‐2 cells following miR‐148a‐3p depletion (Figure 3D and Figure S1A). Conversely, miR‐148a‐3p overexpression increased the expression of E‐cadherin, accompanying decreased N‐cadherin and vimentin expression (Figure 3D and Figure S1A). Immunofluorescence analysis further confirmed those findings (Figure S2A). Additionally, PC cell morphology was drastically altered by miR‐148a‐3p overexpression or depletion: Mia PaCa‐2 cells in miR‐148a‐3p group had a round or cobblestone‐like appearance, whereas Capan‐2 cells in anti‐miR‐148a‐3p group had a spindle shape as compared with the control cells (Figure S2B). On the basis of these findings, we concluded that miR‐148a‐3p suppresses cell invasion and migration of PC cells in vitro through inhibiting the EMT process.

### 3.4 miR‐148a‐3p inhibits stemness properties of PC cells

**FIGURE 4:**
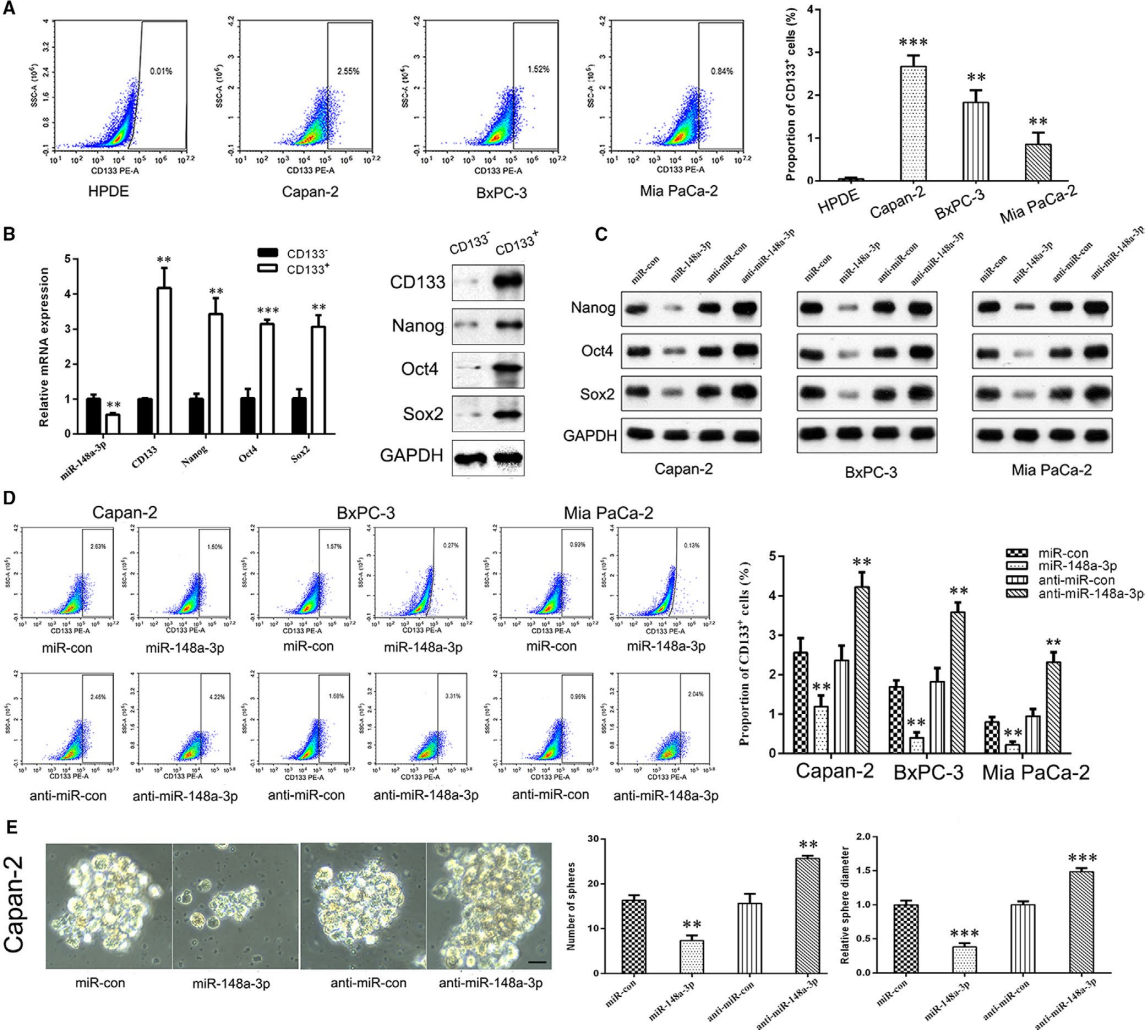
miR‐148a‐3p inhibits the stemness properties of PC cells. A, Flow cytometry analysis of the proportion of CD133^+^ cells in normal HPDE cell line and three PC cell lines (Capan‐2, BxPC‐3 and Mia PaCa‐2). B, The mRNA and protein levels of CD133, Nanog, Oct4 and Sox2 in CD133^+^ and CD133^−^ cells were detected using qRT‐PCR and Western blot analysis, respectively. C, The protein levels of Nanog, Oct4 and Sox2 expression in Capan‐2, BxPC‐3 and Mia PaCa‐2 cells after miR‐148a‐3p up‐regulation or depletion were detected using Western blot analysis. D, Flow cytometry analysis of the proportion of CD133^+^ cells in Capan‐2, BxPC‐3 and Mia PaCa‐2 cells after miR‐148a‐3p up‐regulation or depletion. E, Sphere formation assay was conducted to estimate the effects of miR‐148a‐3p on the cell stemness properties of Capan‐2 cells (400×). Scale bars, 25 μm. Data were expressed as means ± SD of three independent experiments. **P < .01, ***P < .001

As CD133 was well‐recognized as a putative CSC marker for most prevalent solid human cancers including PC,^29^ we detected the proportion of CD133^+^ cells in normal HPDE cell line and three PC cell lines (Capan‐2, BxPC‐3 and Mia PaCa‐2) by using flow cytometry. As shown in Figure 4A, no significant expression of CD133 was seen in normal HPDE cells, but an elevated proportion of CD133^+^ cells was seen in Capan‐2, BxPC‐3 and Mia PaCa‐2 cells. As Capan‐2 cells exhibited higher CD133^+^ proportion cells, we detected the expression of miR‐148a‐3p and stemness‐associated markers in CD133^+^ and CD133^−^ cells isolated from Capan‐2 cells. As shown in Figure 4B, compared to CD133^−^ cells, miR‐148a‐3p was markedly down‐regulated in CD133^+^ cells, whereas the stemness‐associated markers CD133, Nanog, Oct4 and Sox2 were up‐regulated. Furthermore, miR‐148a‐3p overexpression markedly down‐regulated the expression of Nanog, Oct4 and Sox2 in Capan‐2, BxPC‐3 and Mia PaCa‐2 cells, while miR‐148a‐3p depletion up‐regulated these stemness‐associated markers (Figure 4C and Figure S1B). Flow cytometry results also showed that the CD133^+^ proportion cells in Capan‐2, BxPC‐3 and Mia PaCa‐2 cells were significantly reduced by miR‐148a‐3p overexpression, while miR‐148a‐3p depletion caused an opposite effect (Figure 4D). In addition, functional assays demonstrated that miR‐148a‐3p overexpression suppressed the sphere formation ability, while miR‐148a‐3p depletion improved the sphere formation ability of Capan‐2 cells (Figure 4E).

**FIGURE 5:**
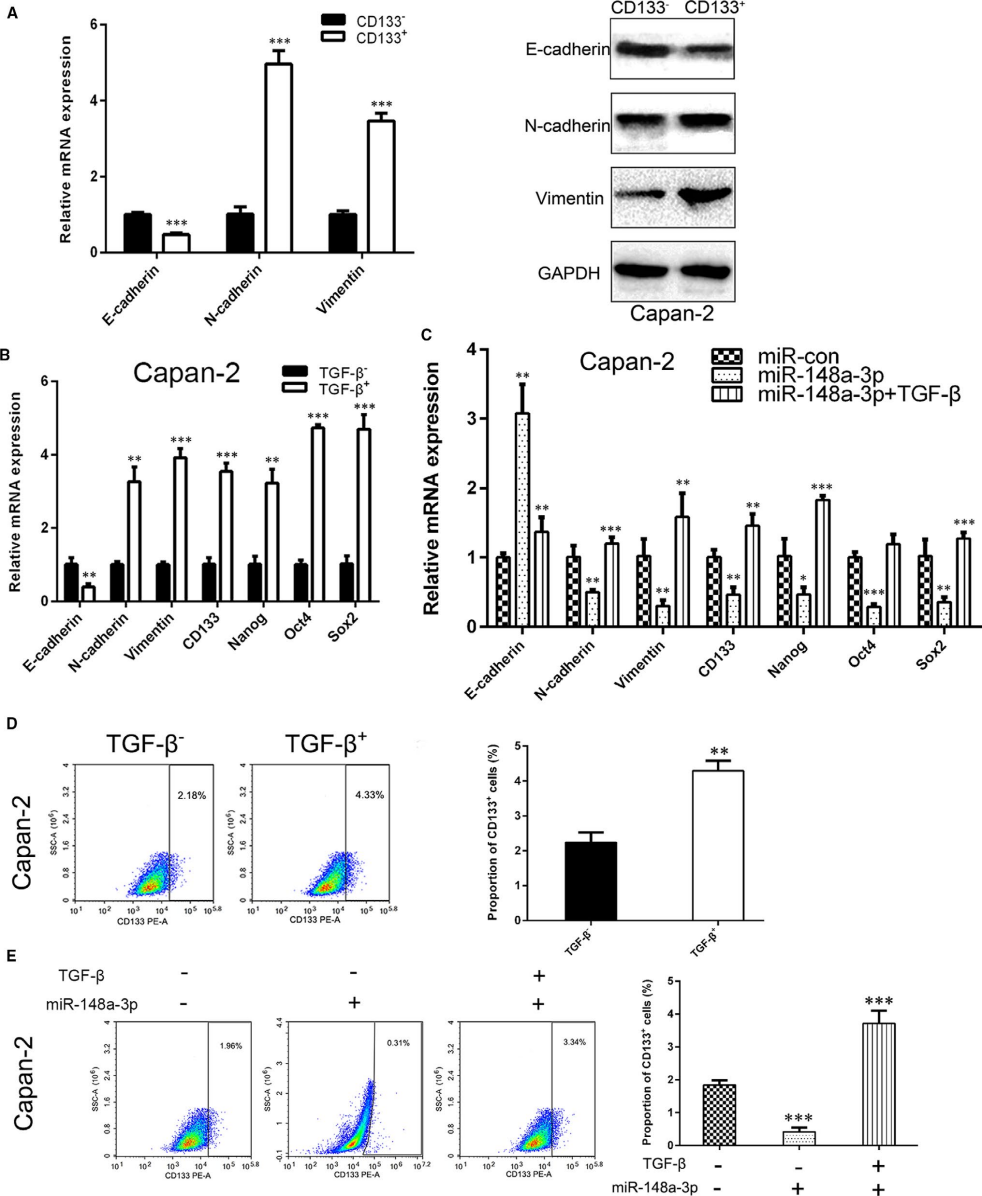
TGF‐β promotes the stemness properties of PC cells. A, The mRNA and protein levels of E‐cadherin, N‐cadherin and vimentin in CD133^+^ and CD133^−^ cells were detected using RT‐qPCR and Western blot analysis, respectively. B, The mRNA levels of E‐cadherin, N‐cadherin, vimentin, CD133, Nanog, Oct4 and Sox2 in Capan‐2 cells after TGF‐β treatment were detected using RT‐qPCR analysis. C, The mRNA levels of E‐cadherin, N‐cadherin, vimentin, CD133, Nanog, Oct4 and Sox2 in Capan‐2 cells after miR‐148a‐3p up‐regulation combine TGF‐β treatment or not were detected using RT‐qPCR analysis. D, Flow cytometry analysis of the proportion of CD133^+^ cells in Capan‐2 cells after TGF‐β treatment. E, Flow cytometry analysis of the proportion of CD133^+^ cells in Capan‐2 cells after miR‐148a‐3p up‐regulation combines TGF‐β treatment or not. Data were expressed as means ± SD of three independent experiments. *P < .05, **P < .01, ***P < .001

Next, we investigated the potential regulatory mechanisms of miR‐148a‐3p on the stemness properties of PC cells. Emerging evidence suggested that EMT‐type cells share many biological characteristics with cancer stem‐like cells, and cancer cells may obtain CSC‐like properties through the EMT process.^30^, ^31^ In this study, we compared the expression of EMT markers between CD133^+^ and CD133^−^ Capan‐2 cells. The results showed that both mRNA and protein levels of mesenchymal markers N‐cadherin and vimentin were up‐regulated, whereas epithelial marker E‐cadherin was down‐regulated in CD133^+^ Capan‐2 cells, compared with CD133^−^ Capan‐2 cells (Figure 5A). In addition, the expression level of CSC markers (CD133, Nanog, Oct4 and Sox2) and the proportion of CD133^+^ cells were elevated when PC cells were exposed to the inducer of EMT (TGF‐β) for 48 hours (Figure 5B,D). Furthermore, the reduced mRNA expression of stemness‐associated markers and the decreased proportion of CD133^+^ cells caused by miR‐148a‐3p overexpression were partly restored by TGF‐β treatment in PC cells (Figure 5C,E). Collectively, these results confirmed the involvement of EMT process in the regulation of miR‐148a‐3p on stemness properties of PC cells.

### 3.5 miR‐148a‐3p directly targets Wnt1‐mediated Wnt/β‐catenin signalling pathway in vitro

**FIGURE 6:**
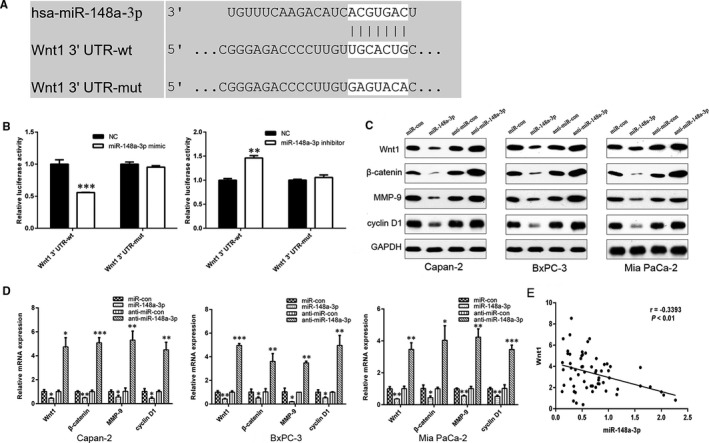
miR‐148a‐3p directly targets Wnt1. A, The predicted binding sites of miR‐148a‐3p in the 3′ UTR of Wnt1 determined by TargetScan. B, Luciferase activities of Wnt1‐3′UTR‐wt and Wnt1‐3′UTR‐mut constructs when transfecting with either miR‐148a‐3p mimics or inhibitors in HEK‐293T cells. C, D, The protein and mRNA levels of Wnt1, β‐catenin, MMP‐9 and cyclin D1 in Capan‐2, BxPC‐3 and Mia PaCa‐2 cells after miR‐148a‐3p up‐regulation or depletion were detected using Western blot and RT‐qPCR analysis, respectively. E. Spearman’s correlation analysis was utilized for the association between miR‐148a‐3p and Wnt1 in PC tissues (*r* = −.3393, *P* < .01). Data were expressed as means ± SD of three independent experiments. *P < .05, **P < .01, ***P < .001

Wnt1 was a potential target of miR‐148a‐3p by bioinformatical prediction tools, including TargetScan 7.2 (http://www.targetscan.org/vert_72/) and miRBase (http://www.mirbase.org/; Figure 6A). A dual‐luciferase reporter assay showed that miR‐148a‐3p overexpression significantly decreased the luciferase activity of the reporter with wild‐type Wnt1‐3′ UTR but unaffected the activity of the mutant‐type vector, and miR‐148a‐3p depletion had the opposite effect (Figure 6B). We also found that miR‐148a‐3p negatively regulated the protein and mRNA levels of Wnt1 in Capan‐2, BxPC‐3 and Mia PaCa‐2 cells (Figure 6C,D). Furthermore, the expression level of miR‐148a‐3p was inversely associated with Wnt1 expression in PC tissues (Figure 6E). These data made it evident that Wnt1 is a direct target of miR‐148a‐3p in PC.

To further investigate whether miR‐148a‐3p could modulate Wnt1‐mediated Wnt/β‐catenin signalling pathway, the expression levels of downstream targets of this pathway including β‐catenin, MMP‐9 and cyclin D1 were detected by using qRT‐PCR and Western blot analysis. The results showed that miR‐148a‐3p overexpression markedly down‐regulated the expression of β‐catenin, MMP‐9 and cyclin D1 in Mia PaCa‐2, BxPC‐3 and Capan‐2 cells, while miR‐148a‐3p depletion up‐regulated the expression of these genes (Figure 6C,D). Thus, these findings indicated that miR‐148a‐3p could inhibit Wnt/β‐catenin signalling pathway by directly targeting Wnt1.

**FIGURE 7:**
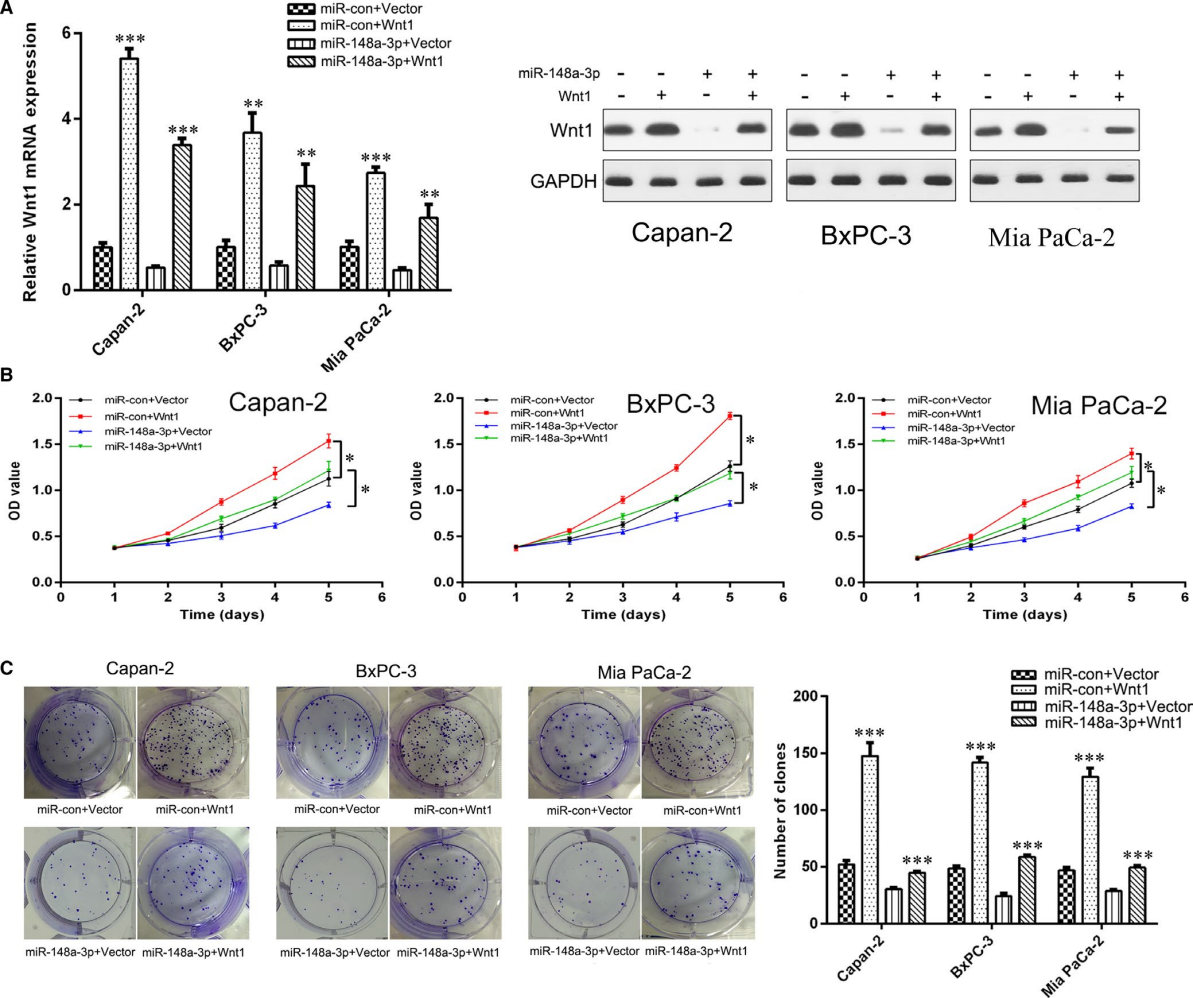
miR‐148a‐3p inhibits PC cell proliferation through targeting Wnt1. A, The mRNA and protein levels of Wnt1 in Capan‐2, BxPC‐3 and Mia PaCa‐2 cells after Wnt1 and/or miR‐148a‐3p up‐regulation were detected using RT‐qPCR and Western blot analysis, respectively. B, C, The proliferation of Capan‐2, BxPC‐3 and Mia PaCa‐2 cells after Wnt1 and/or miR‐148a‐3p up‐regulation was detected using CCK‐8 assay and colony formation assay. Data were expressed as means ± SD of three independent experiments. *P < .05, **P < .01, ***P < .001

**FIGURE 8:**
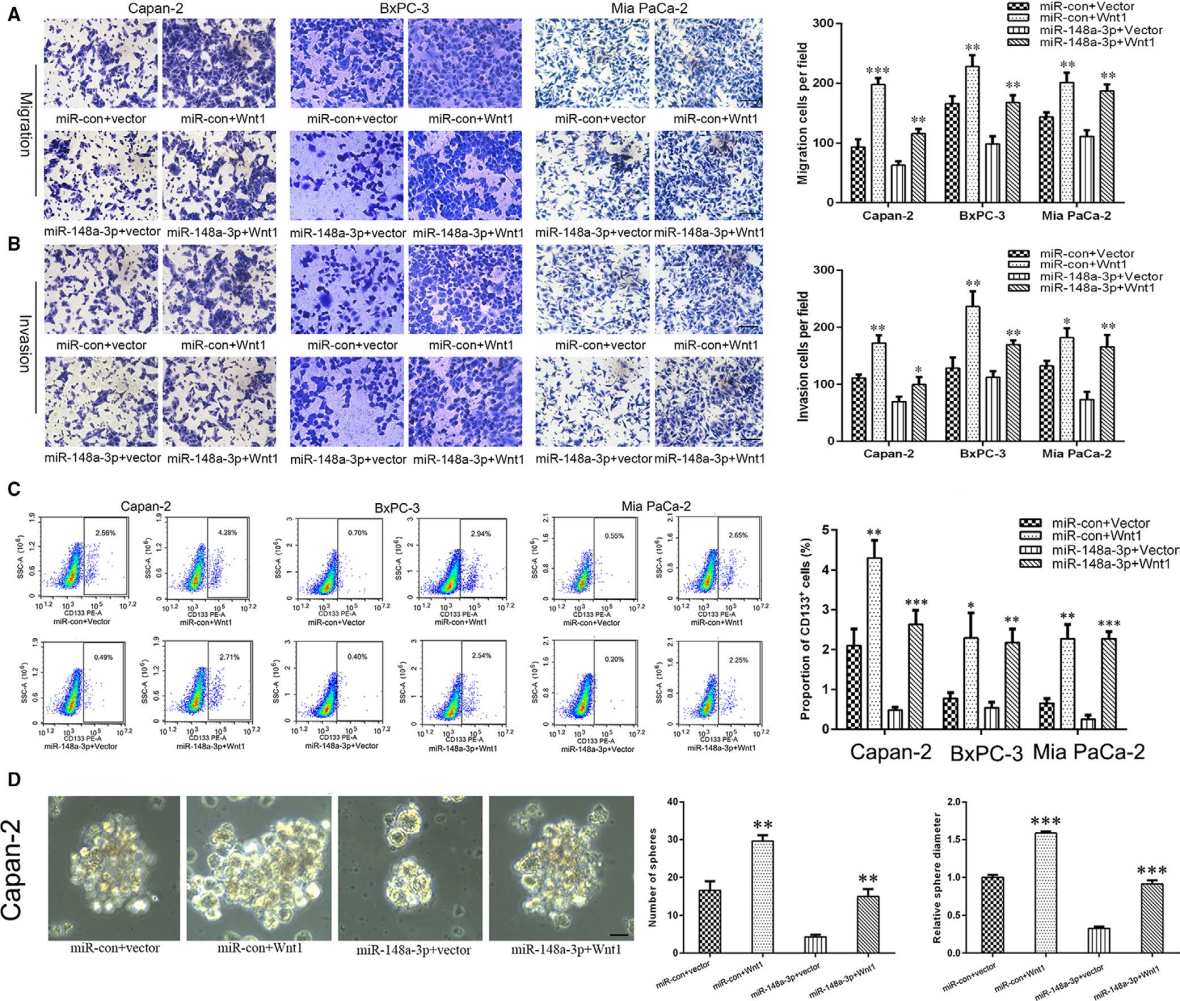
miR‐148a‐3p inhibits PC cell migration, invasion and stemness properties through targeting Wnt1. A,B, The migration and invasion capacities of Capan‐2, BxPC‐3 and Mia PaCa‐2 cells after Wnt1 and/or miR‐148a‐3p up‐regulation were evaluated using Transwell assay (200×). Scale bars, 50 μm. C, Flow cytometry analysis of the proportion of CD133^+^ cells in Capan‐2, BxPC‐3 and Mia PaCa‐2 cells after Wnt1 and/or miR‐148a‐3p up‐regulation. D, Sphere formation assay was conducted to evaluate the stemness properties of Capan‐2 cells after Wnt1 and/or miR‐148a‐3p up‐regulation (400×). Scale bars, 25 μm. Data were expressed as means ± SD of three independent experiments. *P < .05, **P < .01, ***P < .001

Subsequently, Wnt1 siRNA and Wnt1 overexpression plasmid were transfected into PC cells, and the transfection efficacy was confirmed by qRT‐PCR and Western blot analysis (Figure 7A and Figure S3A). We observed that Wnt1 overexpression promoted PC cell proliferation (Figure 7B,C), migration (Figure 8A) and invasion (Figure 8B), while Wnt1 knockdown had the opposite effect (Figure S3B,C). Furthermore, Wnt1 overexpression increased the proportion of CD133^+^ cells in Capan‐2, BxPC‐3 and Mia PaCa‐2 cells (Figure 8C) and promoted the sphere formation ability of Capan‐2 cells (Figure 8D). Correspondingly, Wnt1 knockdown decreased the proportion of CD133^+^ cells in PC cells (Figure S3D). In addition, the ectopic Wnt1 expression effectively reversed the inhibition of proliferation (Figure 7B,C), migration (Figure 8A), invasion (Figure 8B) and stemness properties (Figure 8C,D) of PC cells induced by miR‐148a‐3p overexpression. In summary, miR‐148a‐3p inhibited PC cell proliferation, migration, invasion and stemness properties by targeting Wnt1‐mediated Wnt/β‐catenin signalling pathway in vitro.

### 3.6 miR‐148a‐3p functions in vivo

**FIGURE 9:**
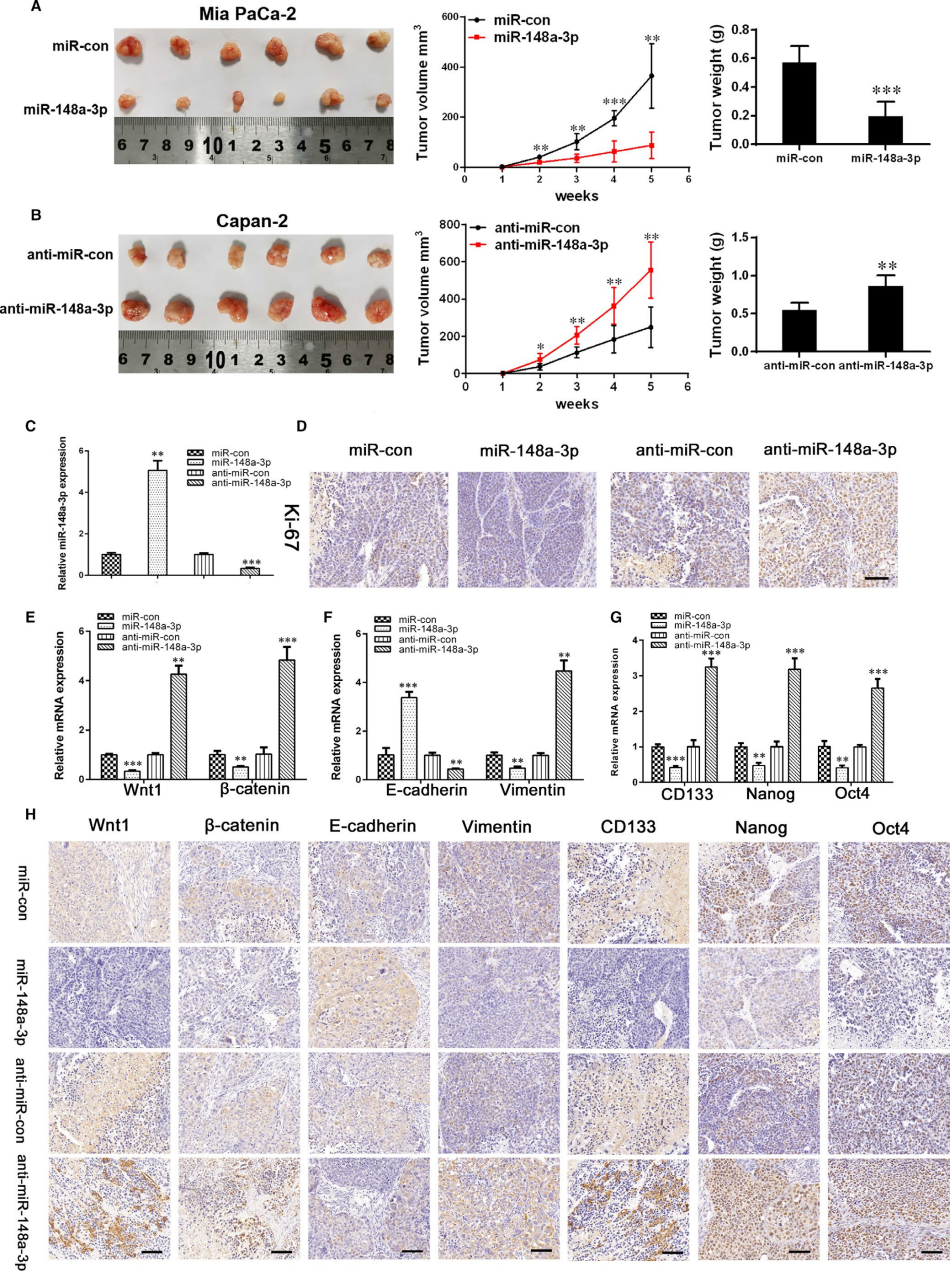
miR‐148a‐3p inhibits tumour growth, EMT and stemness properties in mouse subcutaneous xenograft model. A, Tumour growth curves and tumour weights of miR‐148a‐3p‐stable–expressing Mia PaCa‐2 cell–derived xenografts. B, Tumour growth curves and tumour weights of anti‐miR‐148a‐3p‐stable–expressing Capan‐2 cell–derived xenografts. C, qRT‐PCR analysis of miR‐148a‐3p expression in xenograft tumours. D, Representative immunohistochemistry staining of proliferation‐related molecules Ki‐67 in xenograft tumours (400×). Scale bars, 25 μm. E, qRT‐PCR analysis of Wnt1 and β‐catenin expression in xenograft tumours. F, qRT‐PCR analysis of EMT marker (E‐cadherin and vimentin) expression in xenograft tumours. G, qRT‐PCR analysis of stemness‐associated marker (CD133, Nanog and Oct4) expression in xenograft tumours. H, Representative immunohistochemistry staining of Wnt1, β‐catenin, EMT markers (E‐cadherin and vimentin) and stemness‐associated markers (CD133, Nanog and Oct4) in xenograft tumours (400×). Scale bars, 25 μm. Data were expressed as means ± SD of three independent experiments. *P < .05, **P < .01, ***P < .001

To verify the above findings in vivo, we performed studies using a nude mouse subcutaneous xenograft model. We observed that miR‐148a‐3p‐stable–expressing Mia PaCa‐2 cell–derived xenografts grew at a significantly slower rate, with lower tumour volumes and weight, compared with miR‐con group (Figure 9A). In contrast, the volume and weight of tumours formed by anti‐miR‐148a‐3p‐stable–expressing Capan‐2 cells were elevated compared with those in anti‐miR‐con group (Figure 9B). qRT‐PCR results showed a significantly elevated expression level of miR‐148a‐3p in miR‐148a‐3p‐stable–expressing Mia PaCa‐2 cell–derived xenografts and a markedly decreased expression level of miR‐148a‐3p in anti‐miR‐148a‐3p‐stable–expressing Capan‐2 cell–derived xenografts (Figure 9C). IHC analysis showed the expression level of Ki‐67 in miR‐148a‐3p group was lower than that in miR‐con group, whereas it was higher in anti‐miR‐148a‐3p group than that in anti‐miR‐con group (Figure 9D). We further evaluated the expression of Wnt1, β‐catenin, EMT markers (E‐cadherin and vimentin) and stemness‐associated markers (CD133, Nanog and Oct4) in xenograft tumours. Both the results of qRT‐PCR and IHC analysis confirmed that the expression levels of Wnt1, β‐catenin, vimentin, CD133, Nanog and Oct4 were significantly down‐regulated, whereas the expression level of E‐cadherin was up‐regulated in miR‐148a‐3p group, compared with miR‐con group; conversely, the anti‐miR‐148a‐3p group showed a significantly elevated expression level of Wnt1, β‐catenin, vimentin, CD133, Nanog and Oct‐4, and a markedly decreased expression level of E‐cadherin, compared with anti‐miR‐con group (Figure 9E‐H). Collectively, our subcutaneous xenograft studies provided further evidence that miR‐148a‐3p suppresses the growth, EMT and stemness properties of PC via inhibiting Wnt1/β‐catenin pathway in vivo.

## 4 DISCUSSION

**FIGURE 10:**
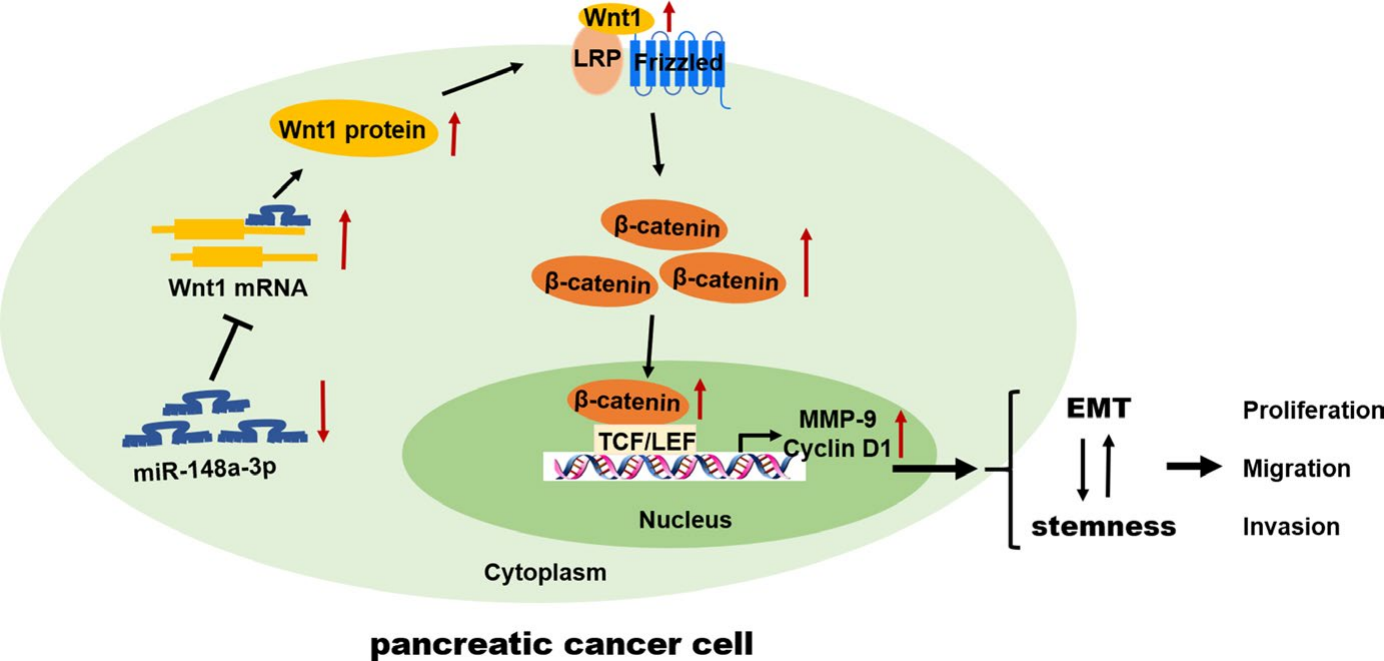
The schematic illustration of the potential molecular mechanism of miR‐148a‐3p as a tumour suppressor in PC. miR‐148a‐3p was remarkably down‐regulated in PC and negatively regulated Wnt1, which was a direct functional target of miR‐148a‐3p. miR‐148a‐3p suppressed EMT and stemness properties as well as the proliferation, migration and invasion via inhibiting Wnt1‐mediated Wnt/β‐catenin pathway in PC

Despite advances in diagnostic and surgical techniques over the past decades, PC still remains one of the most aggressive and lethal malignancies characterized by rapid tumour progression and early metastasis. Dysregulation of miRNAs has been shown in various human malignancies and involved in the initiation, progression and metastasis of cancers.^32^, ^33^ There is increasing evidence, suggesting that decreased miR‐148a‐3p expression results in cancer development. In the present study, miR‐148a‐3p expression was remarkably down‐regulated in PC tissues and cell lines, and negatively correlated with Wnt1 expression. Low expression of miR‐148a‐3p was associated with poorer OS in patients with PC. These findings indicated miR‐148a‐3p could be served as a potential biomarker to predict the prognosis of patients with PC. In vitro and in vivo experiments have shown that miR‐148a‐3p suppressed EMT and stemness properties as well as the proliferation, migration and invasion of PC cells. Wnt1 was first identified as a direct and functional target of miR‐148a‐3p in PC. Moreover, miR‐148a‐3p played its biological functions via inhibiting Wnt1‐mediated Wnt/β‐catenin pathway. We also confirmed that the EMT process is involved in the regulation of miR‐148a‐3p on stemness properties of PC cells (Figure 10).

EMT is a common feature of various types of tumour that allows the epithelial cells to acquire mesenchymal phenotype, resulting in enhanced migration and invasion capacities, leading to metastasis and poor prognosis in multiple solid cancers.^11^, ^14^ As the characteristic of EMT, cancer cells gradually lose expression of epithelial markers, such as E‐cadherin, whereas some mesenchymal marker expressions are increased including vimentin, N‐cadherin, zinc finger E‐box‐binding homeobox 1 (ZEB1) and ZEB2.^11^ Increasing evidence has demonstrated that miRNAs play a key role in the regulation of the EMT process in PC, such as miR‐15a,^34^ miR‐330‐5p^35^ and miR‐200a.^36^ Here, we found that miR‐148a‐3p overexpression increased the expression of E‐cadherin, accompanying with decreased N‐cadherin and vimentin expression in PC cells; while miR‐148a‐3p depletion had an opposite effect. Additionally, cell morphology was drastically altered by miR‐148a‐3p overexpression or depletion. These findings are in agreement with the inhibitory effects of miR‐148a‐3p in the migration and invasion of PC cell. Therefore, miR‐148a‐3p suppressed cell invasion and migration of PC cells through inhibiting the EMT process.

CSCs play critical roles in tumour progression, distant metastasis and therapeutic resistance.^15^, ^17^, ^18^ In 2007, Li et al^37^ firstly identified pancreatic CSCs using CD44 (+)CD24(+)ESA(+) as surface markers. Since then, pancreatic CSCs have been recognized in succession by using various surface markers, including CD133,^29^ CD44,^38^ CD24, c‐met and ALDH.^39^ In the present study, we identified pancreatic CSCs using CD133 as a surface marker and found that the proportion of CSCs in PC cells was significantly elevated as to normal HPDE cells. To date, many miRNAs have been identified to function as stemness maintenance in PC.^36^, ^40^ miR‐148a has been confirmed to be involved in the maintenance of CSC‐like properties of hepatocellular carcinoma.^20^, ^21^ Li et al^38^ demonstrated that miR‐148a‐3p is dramatically decreased in breast cancer stem cells and could be regulated by HOTTIP to suppress the stemness of breast cancer stem cells. Similarly, our data showed that miR‐148a‐3p was significantly down‐regulated, whereas the CSC markers, including CD133, Nanog, Oct4 and Sox2, were up‐regulated in pancreatic CSCs. More importantly, the proportion of CSCs, the expression of CSC markers and the sphere formation ability of PC cells could be regulated by miR‐148a‐3p. Collectively, these findings indicated that miR‐148a‐3p played a critical role in the maintenance of stemness properties in PC. EMT‐type cells share many biological characteristics with cancer stem‐like cells, and the EMT process may give rise to CSCs or at least cells with stem cell–like properties. Jin et al^41^ showed that let‐7a inhibits sphere formation efficiency of hepatocellular carcinoma cells through alleviating EMT. Moreover, the inducer of EMT (Snail and TGF‐β) contributes to the maintenance of stem cell–like properties of PC cell.^42^, ^43^ Overlapping of these two properties implies that they may share similar molecules/pathways. In the present study, TGF‐β promoted the stemness properties of PC cells, partly restored the inhibitory effect of miR‐148a‐3p on the stemness properties of PC cells. These findings confirmed that the EMT process is involved in the regulation of miR‐148a‐3p on stemness properties of PC cells.

It is well‐known that miRNAs perform the specific functions through inhibiting the target mRNAs expression.^44^ More than 800 genes were predicted as potential targets gene of miR‐148a‐3p by bioinformatical prediction tools TargetScan and miRBase. Among these potential targets, Wnt10b,^9^ DNMT1,^10^ CDC25B^45^ and ErbB3^46^ have been identified as direct targets of miR‐148a in PC. In the present study, we focused on Wnt1, an activator of Wnt/β‐catenin pathway, which may be a candidate target of miR‐148a‐3p. Then, the luciferase reporter assay confirmed that miR‐148a‐3p regulated Wnt1 by directly binding its 3'UTR. Functional assays demonstrated that Wnt1 knockdown inhibited the proliferation, migration, invasion and stemness properties of PC cells. Furthermore, the effects of ectopic miR‐148a‐3p on PC cells were rescued by Wnt1 overexpression. Wnt/β‐catenin signalling pathway, a type of canonical Wnt pathway, has been reported to contribute to the EMT process and the maintenance of CSC‐like properties. Liu et al^47^ demonstrated that miR‐504 suppresses the EMT process of glioblastoma via targeting the FZD7‐mediated Wnt/β‐catenin pathway. Jin et al^41^ revealed that let‐7 inhibits the stemness properties of hepatocellular through regulating the EMT and the Wnt signalling pathway. Also, miR‐148a inhibits the EMT process and cancer stem cell–like properties of hepatocellular carcinoma through inhibiting the Wnt signalling pathway.^20^ In the present study, the expression of downstream genes of Wnt/β‐catenin signalling, including β‐catenin, MMP‐9 and cyclin D1, was regulated by miR‐148a‐3p expression. Consistently, the in vivo data showed miR‐148a‐3p inhibits the expression of Wnt1 and β‐catenin. Collectively, these findings indicated that miR‐148a‐3p could exert its biological function through modulating Wnt1‐mediated Wnt/β‐catenin signalling pathway in PC.

In summary, our results show that miR‐148a‐3p expression is remarkably reduced in PC, and its low expression is associated with poorer OS in patients with PC. Furthermore, we confirm that miR‐148a‐3p suppresses proliferation, invasion, EMT and stemness properties of PC cells via directly targeting Wnt1‐mediated Wnt/β‐catenin pathway. These findings provide new evidence for understanding the function and molecular mechanism of miR‐148a‐3p in PC, highlighting the potential of miR‐148a‐3p as a useful prognostic biomarker as well as a therapeutic target for PC.

## CONFLICT OF INTEREST

The authors declare that there is no conflict of interest.

## AUTHOR CONTRIBUTIONS

**Xiaowei Fu:** Data curation (supporting); formal analysis (equal); investigation (equal); methodology (equal); software (equal); visualization (equal); writing‐original draft (equal); writing‐review & editing (equal). **Le Hong:** Formal analysis (equal); investigation (equal); methodology (equal); resources (equal); software (equal); validation (equal); visualization (equal). **Zhengjiang Yang:** Investigation (equal); methodology (equal); resources (equal); software (equal); validation (equal); visualization (equal). **Yi Tu:** Formal analysis (equal); methodology (equal); resources (equal); validation (equal). **Wanpeng Xin:** Investigation (equal); methodology (equal); resources (equal); software (equal). **Ming Zha:** Investigation (equal); methodology (equal); resources (equal); validation (equal). **Shuju Tu:** Investigation (equal); methodology (equal); software (equal); validation (equal). **Gen Sun:** Investigation (equal); methodology (equal); software (equal). **Yong Li:** Resources (equal); supervision (supporting); visualization (equal). **Weidong Xiao:** Conceptualization (equal); data curation (lead); formal analysis (equal); funding acquisition (equal); project administration (equal); supervision (equal); writing‐original draft (equal); writing‐review & editing (equal).

## Supporting information

Fig S1

Fig S2

Fig S3

## Data Availability

The data that support the findings of this study are available from the corresponding author upon reasonable request.
