# Supplementary figures and images for: MicroRNA‐148a‐3p suppresses epithelial‐to‐mesenchymal transition and stemness properties via Wnt1‐mediated Wnt/β‐catenin pathway in pancreatic cancer
Source: J Cell Mol Med. 2020 Oct 7;24(22):13020–35. doi: 10.1111/jcmm.15900 (PMC7701524; doi:10.1111/jcmm.15900)

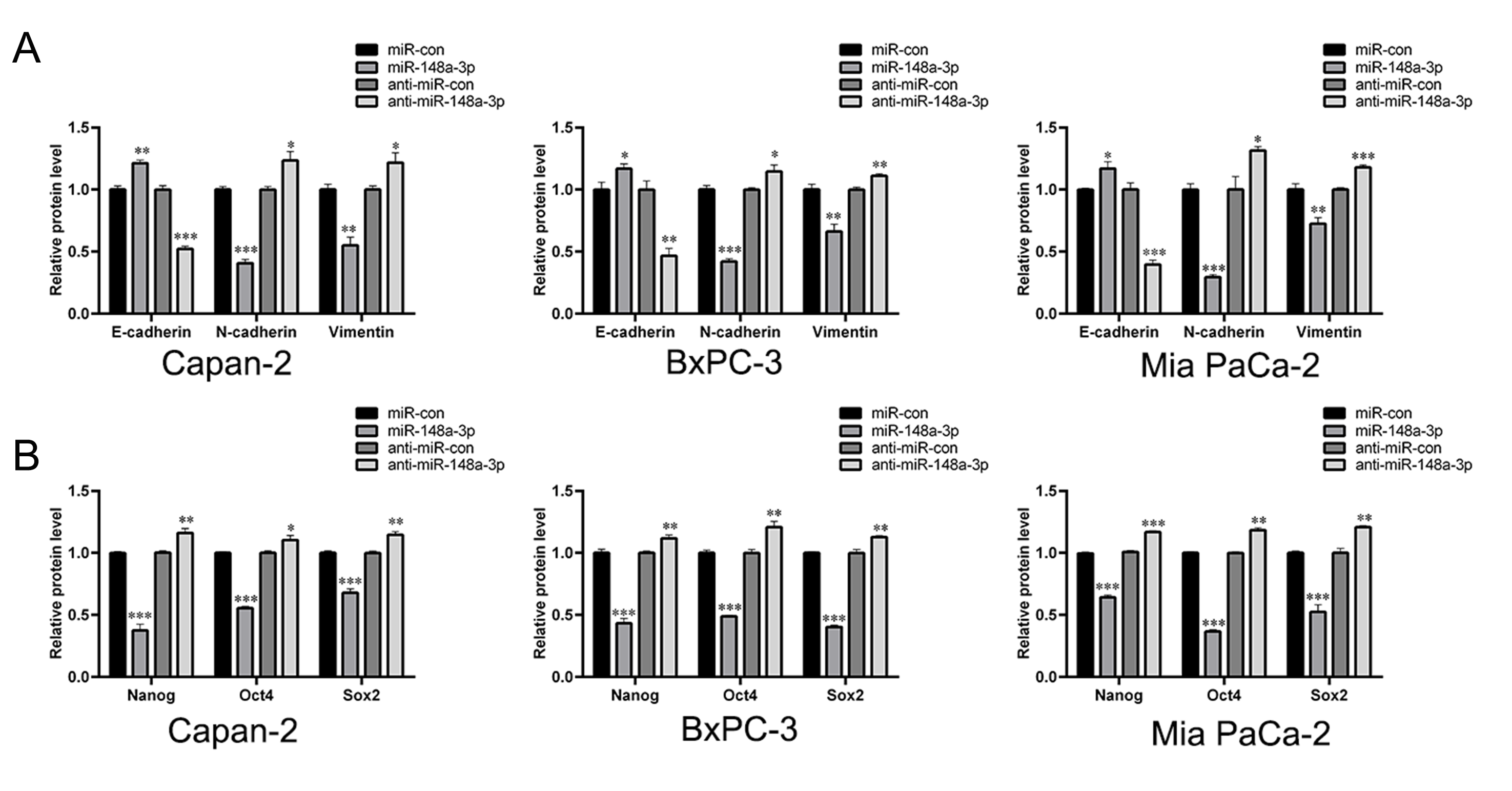

Supplement: Supplementary file 1 — Fig S1 [file JCMM-24-13020-s001.tif]

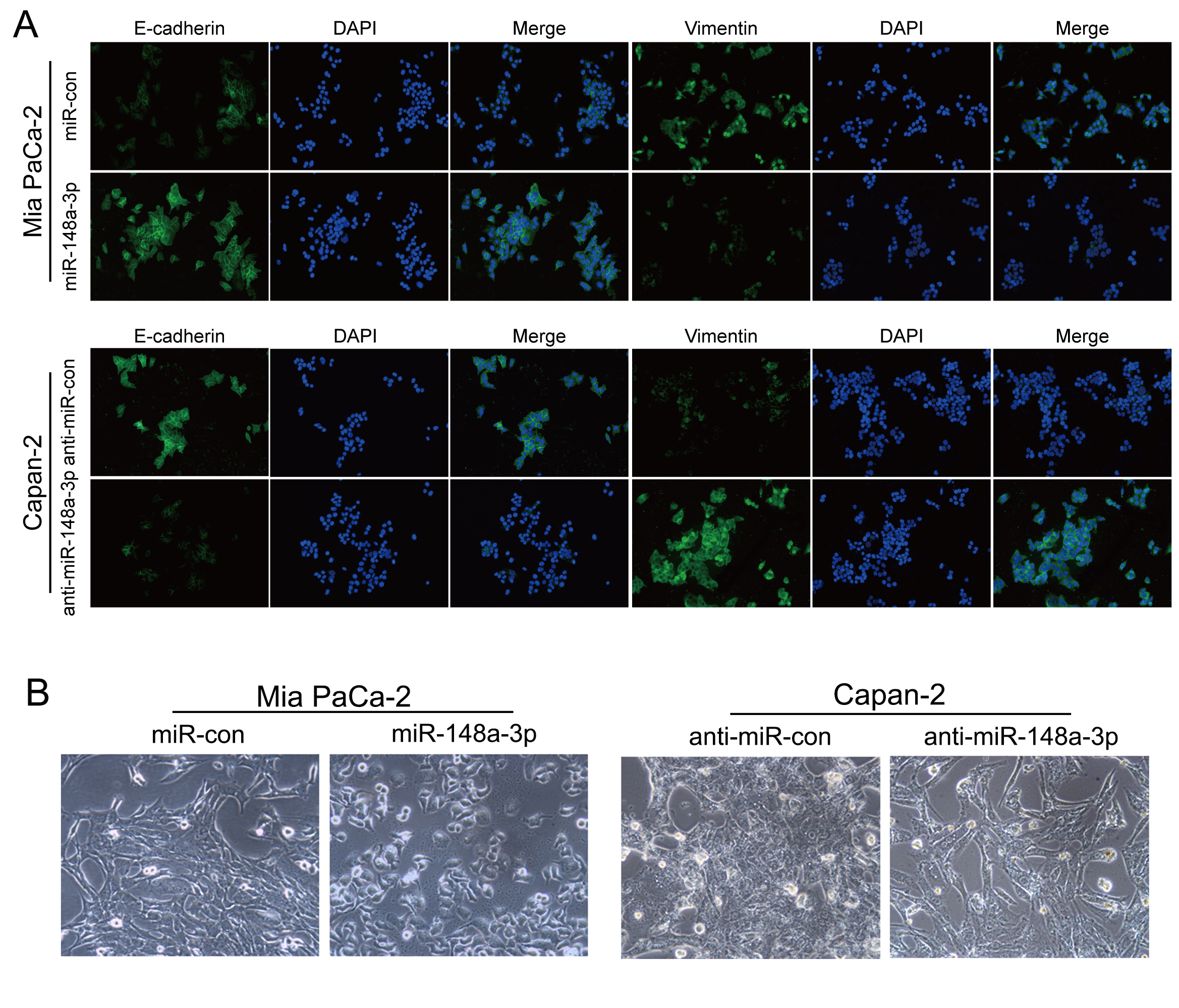

Supplement: Supplementary file 2 — Fig S2 [file JCMM-24-13020-s002.tif]

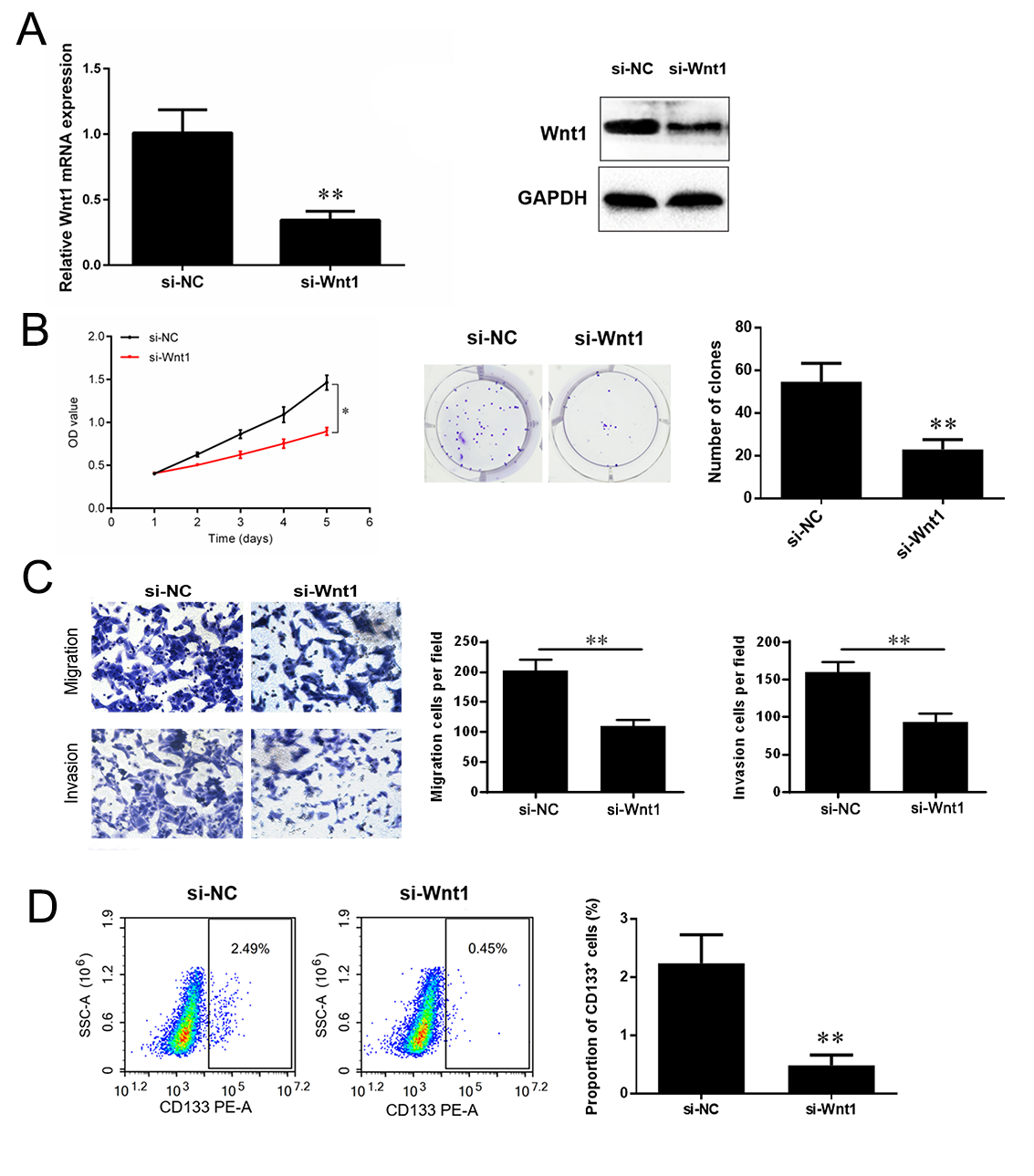

Supplement: Supplementary file 3 — Fig S3 [file JCMM-24-13020-s003.tif]
